# Supplementary material for: Comprehensive Map of the Regulated Cell Death Signaling Network: A Powerful Analytical Tool for Studying Diseases
Source: Cancers (Basel). 2020 Apr 17;12(4):990. doi: 10.3390/cancers12040990 (PMC7226067; doi:10.3390/cancers12040990)
Supplement: Supplementary file 1 [file cancers-12-00990-s001.zip › cancers-744176-supplementary-final/Supplementary Figures.pptx]

## Slide 1
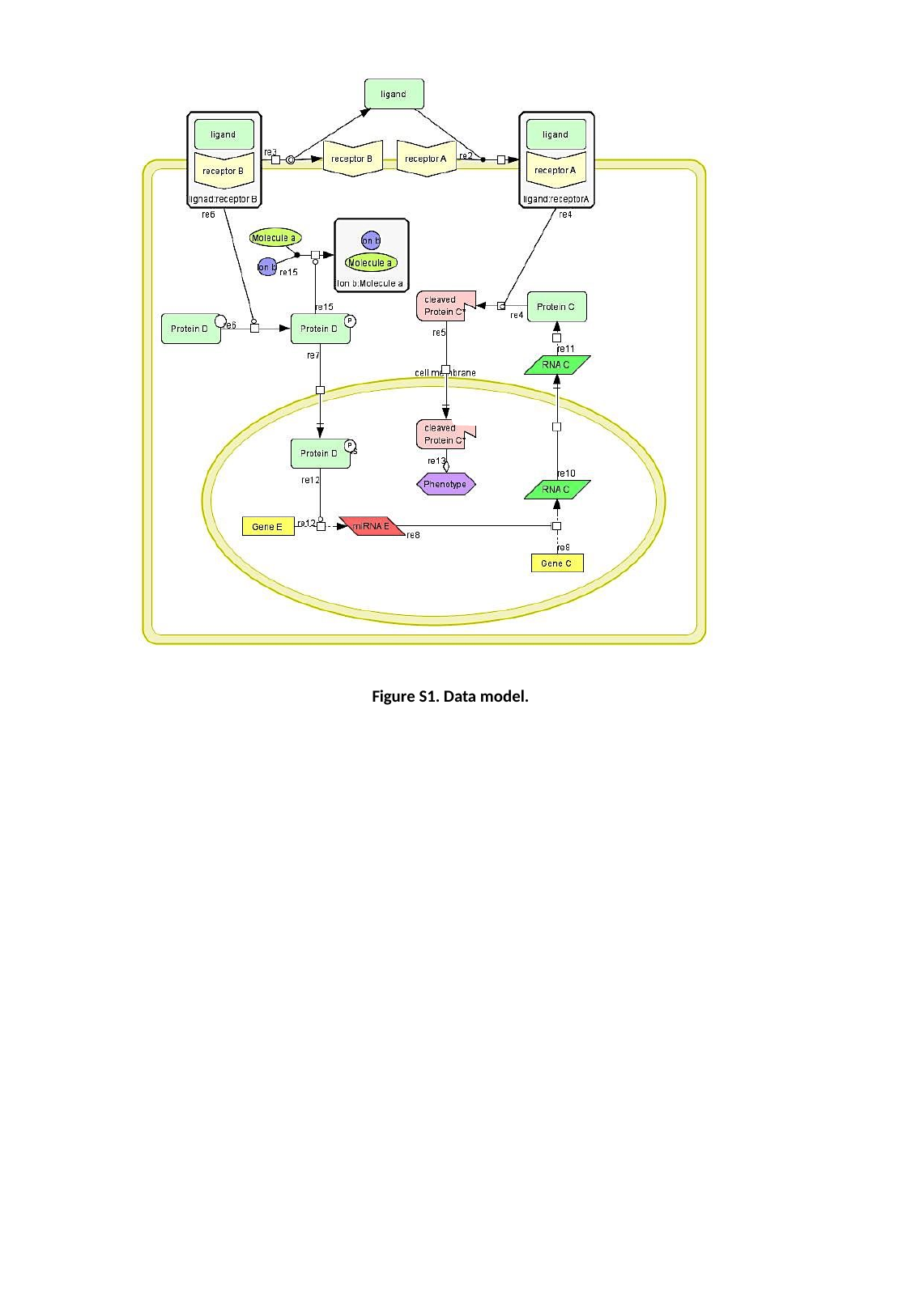

Figure S1. Data model.

## Slide 2
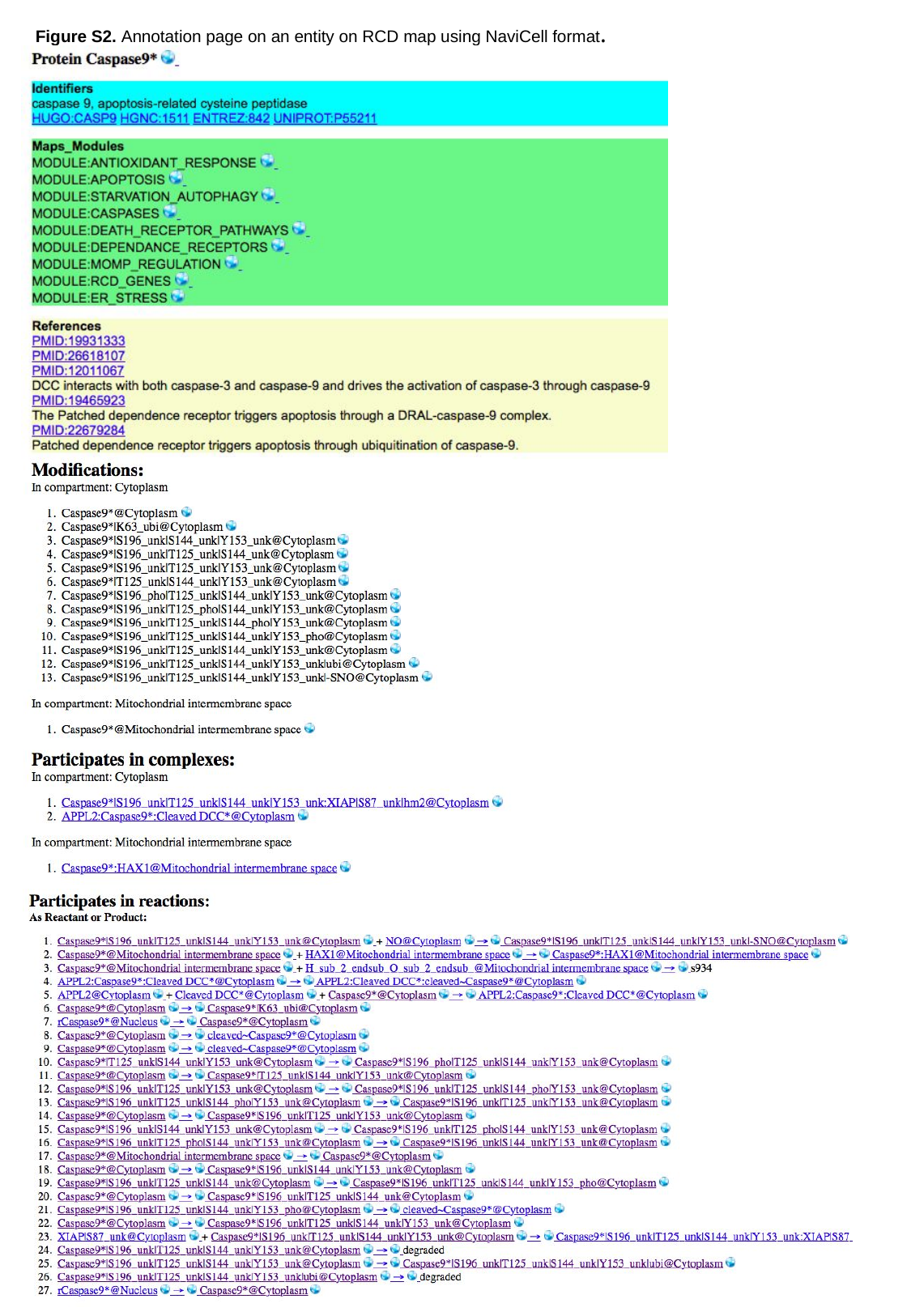

Figure S2. Annotation page on an entity on RCD map using NaviCell format.

## Slide 3
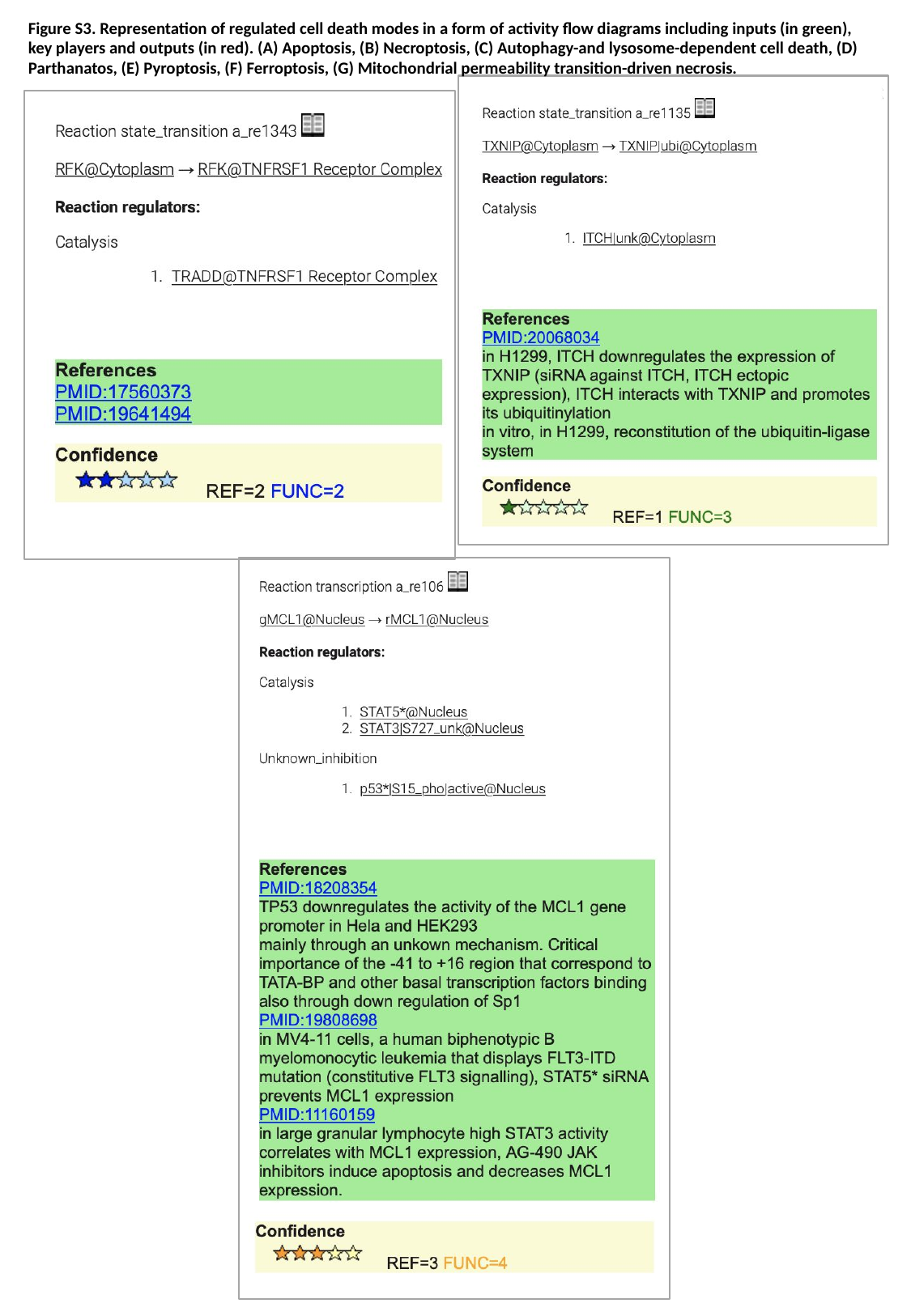

Figure S3. Representation of regulated cell death modes in a form of activity flow diagrams including inputs (in green), key players and outputs (in red). (A) Apoptosis, (B) Necroptosis, (C) Autophagy-and lysosome-dependent cell death, (D) Parthanatos, (E) Pyroptosis, (F) Ferroptosis, (G) Mitochondrial permeability transition-driven necrosis.

## Slide 4
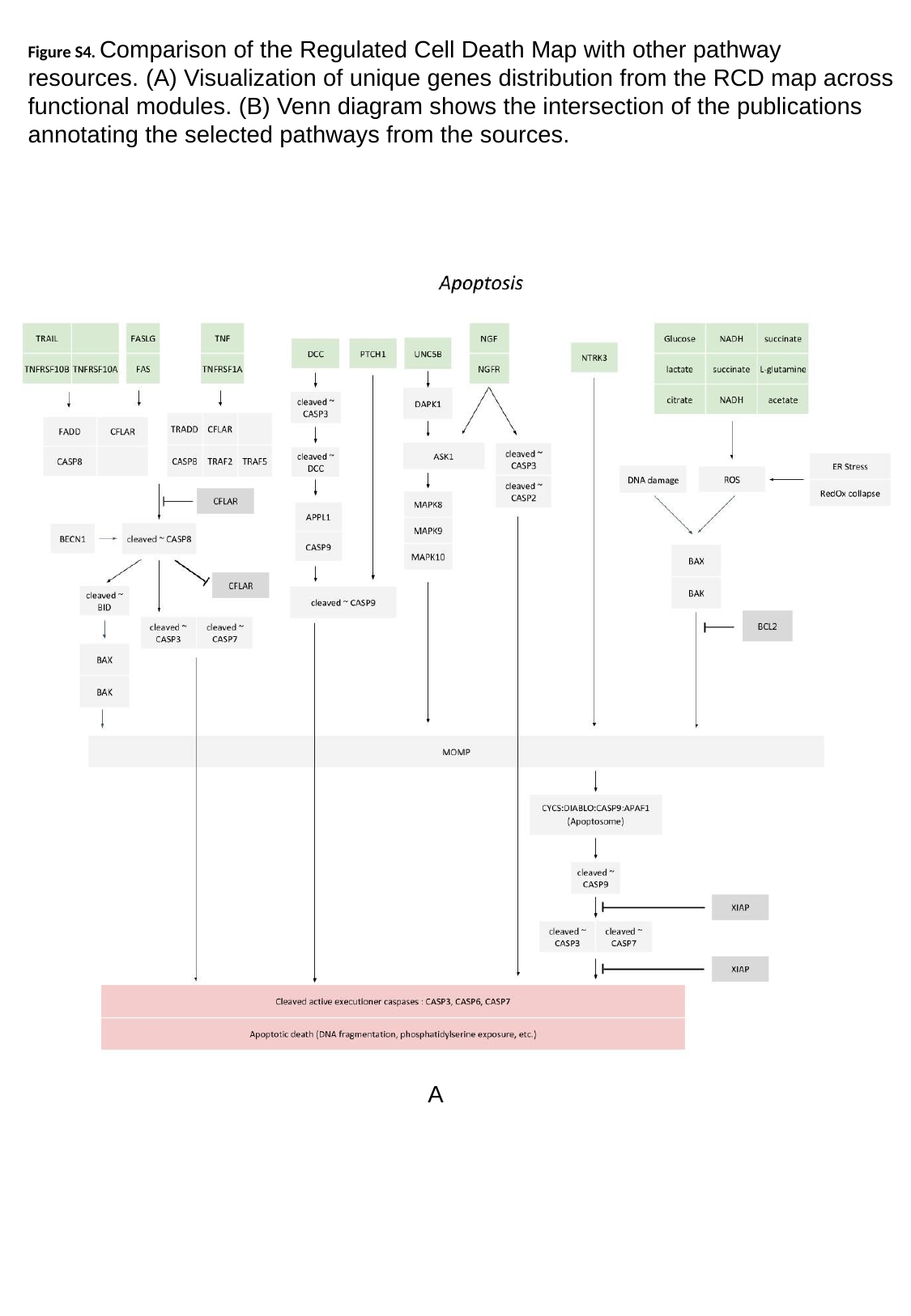

Figure S4. Comparison of the Regulated Cell Death Map with other pathway resources. (A) Visualization of unique genes distribution from the RCD map across functional modules. (B) Venn diagram shows the intersection of the publications annotating the selected pathways from the sources.
A

## Slide 5
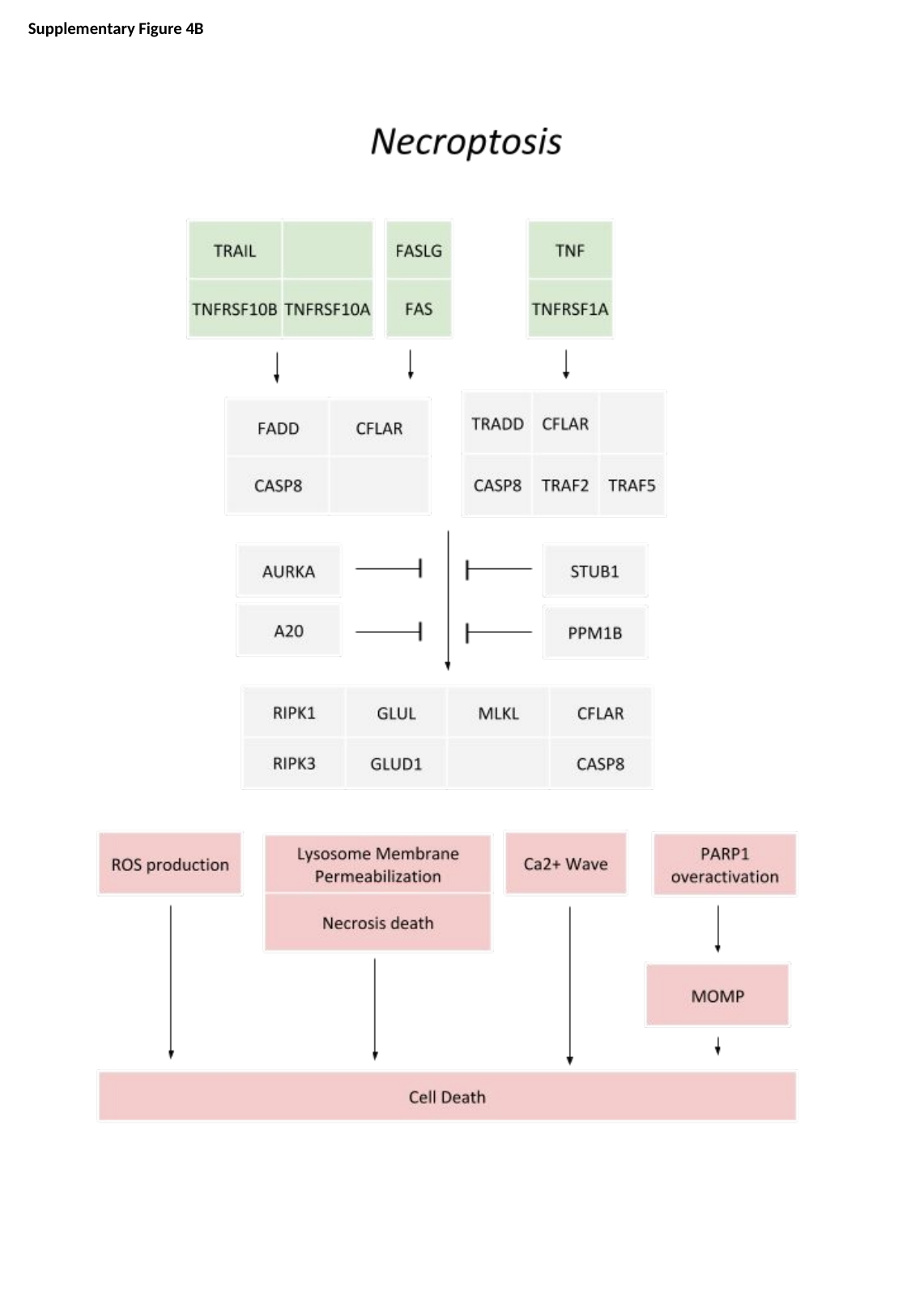

Supplementary Figure 4B

## Slide 6
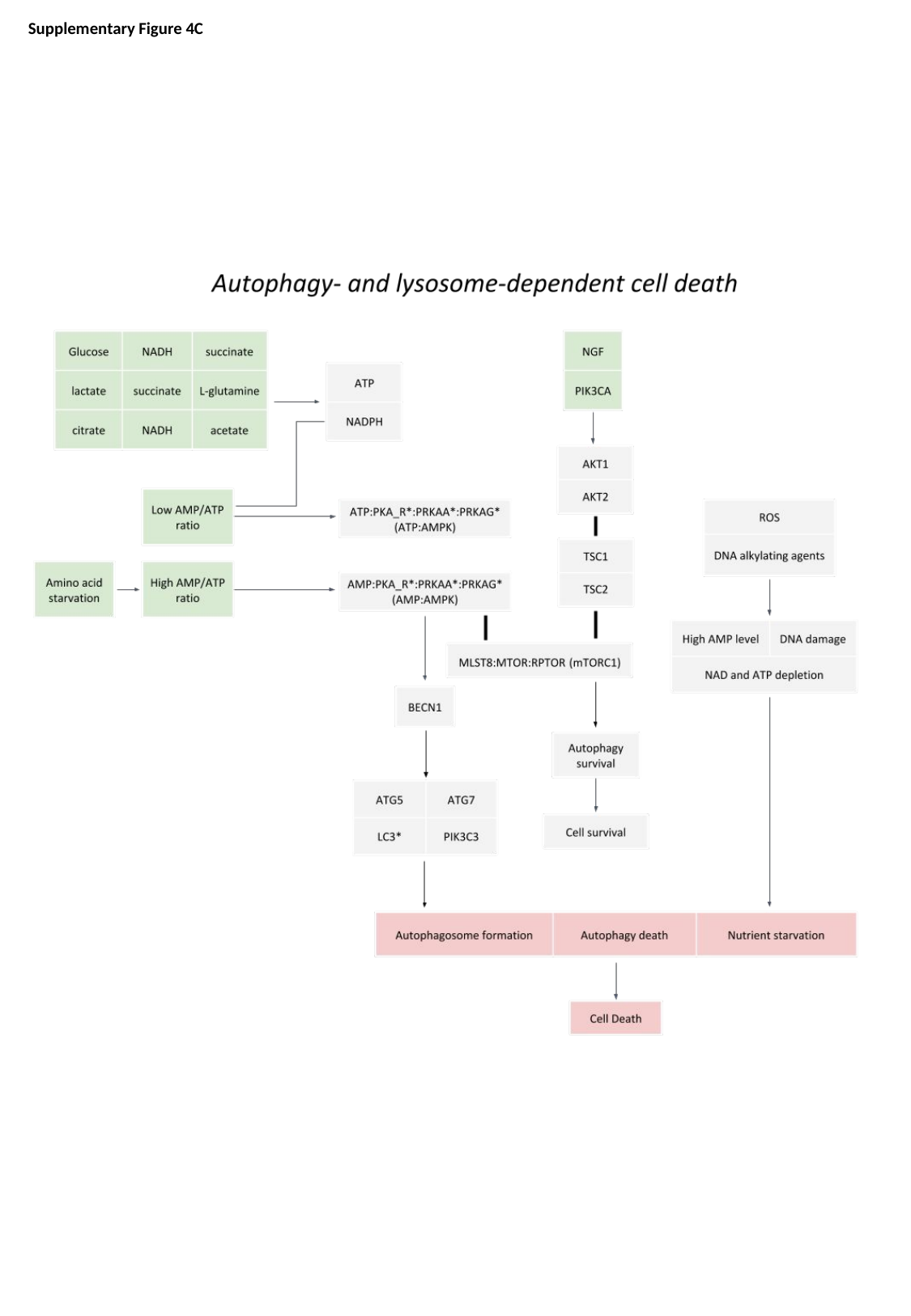

Supplementary Figure 4C

## Slide 7
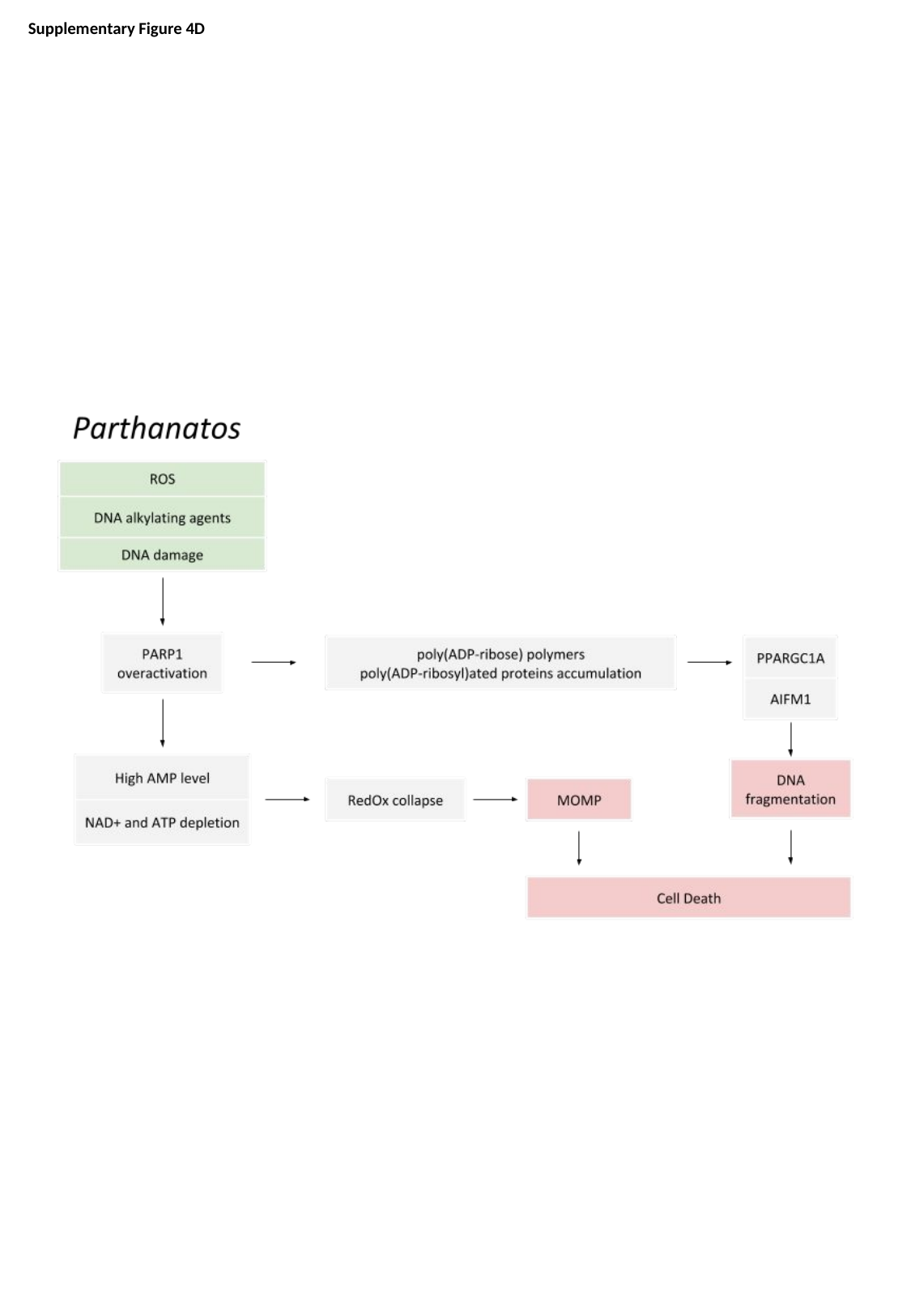

Supplementary Figure 4D

## Slide 8
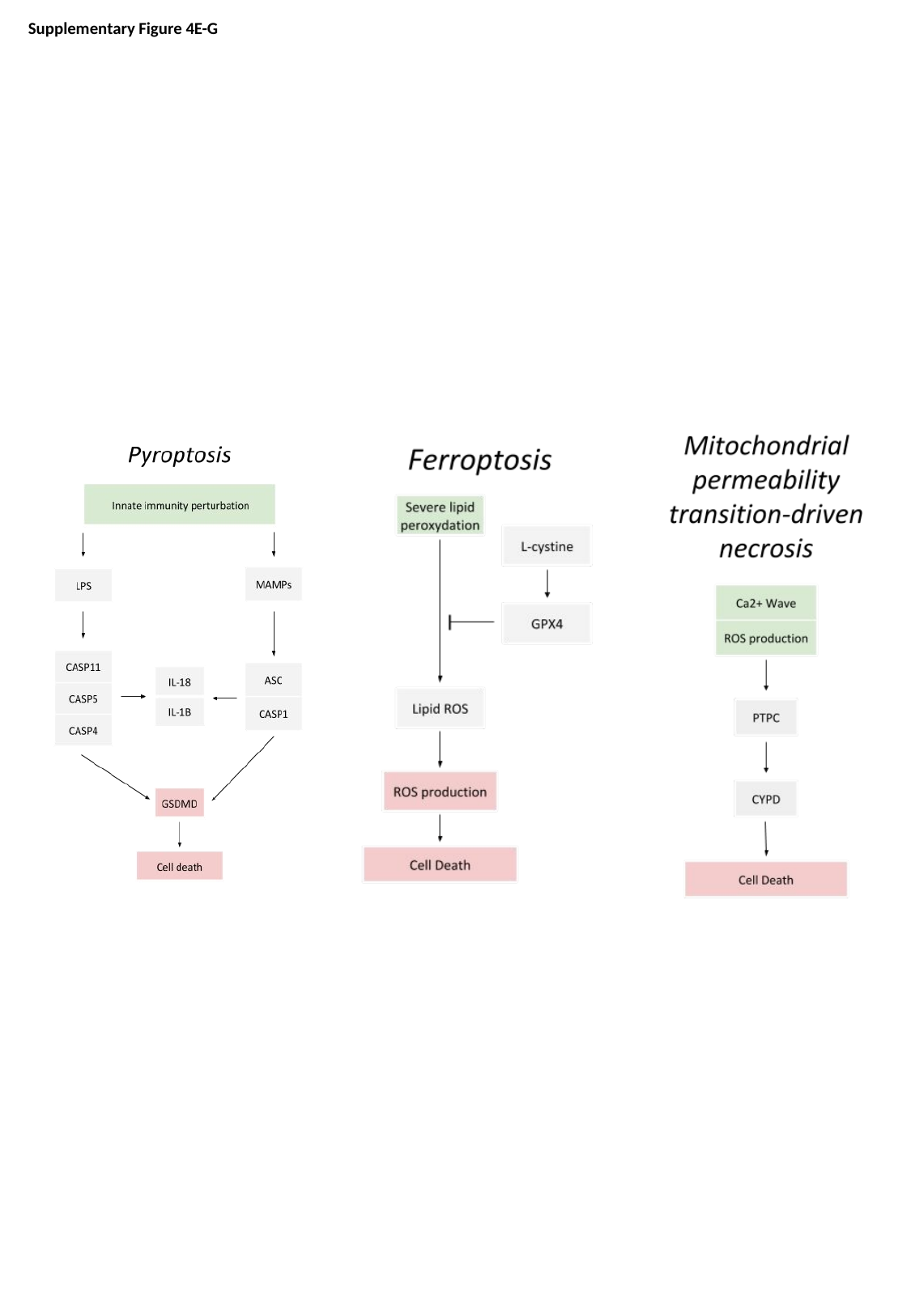

Supplementary Figure 4E-G

## Slide 9
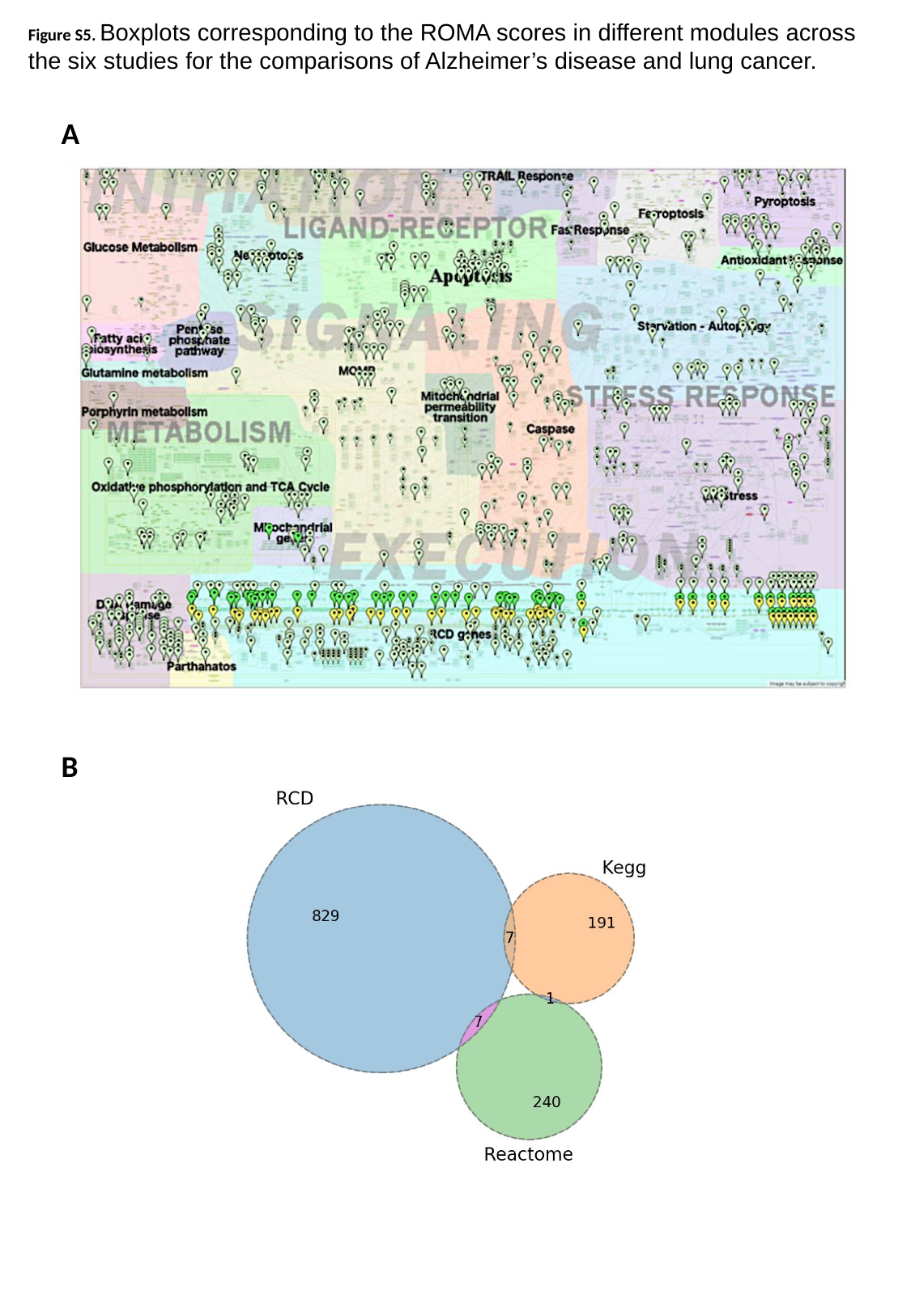

Figure S5. Boxplots corresponding to the ROMA scores in different modules across the six studies for the comparisons of Alzheimer’s disease and lung cancer.
A
B

## Slide 10
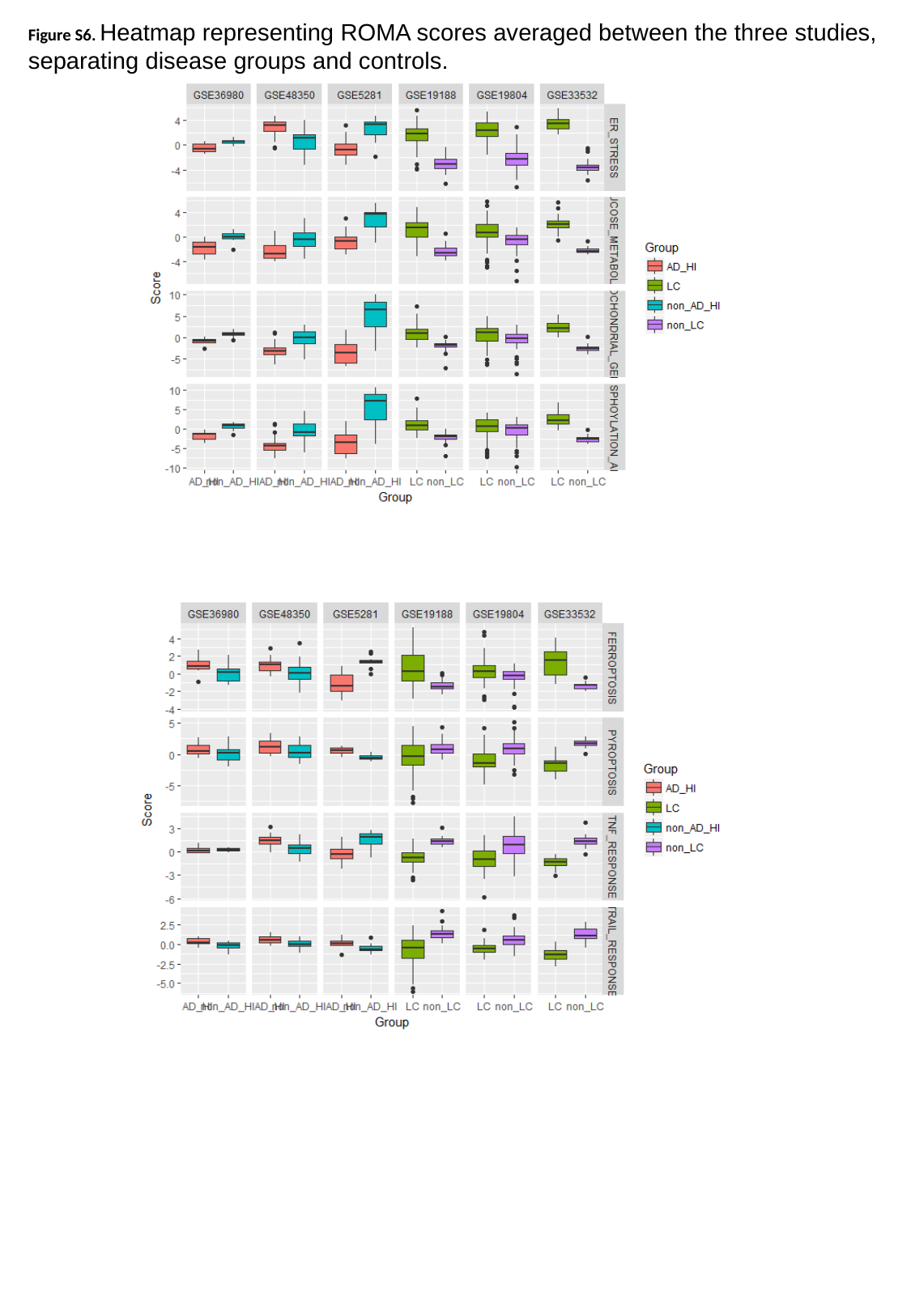

Figure S6. Heatmap representing ROMA scores averaged between the three studies, separating disease groups and controls.

## Slide 11
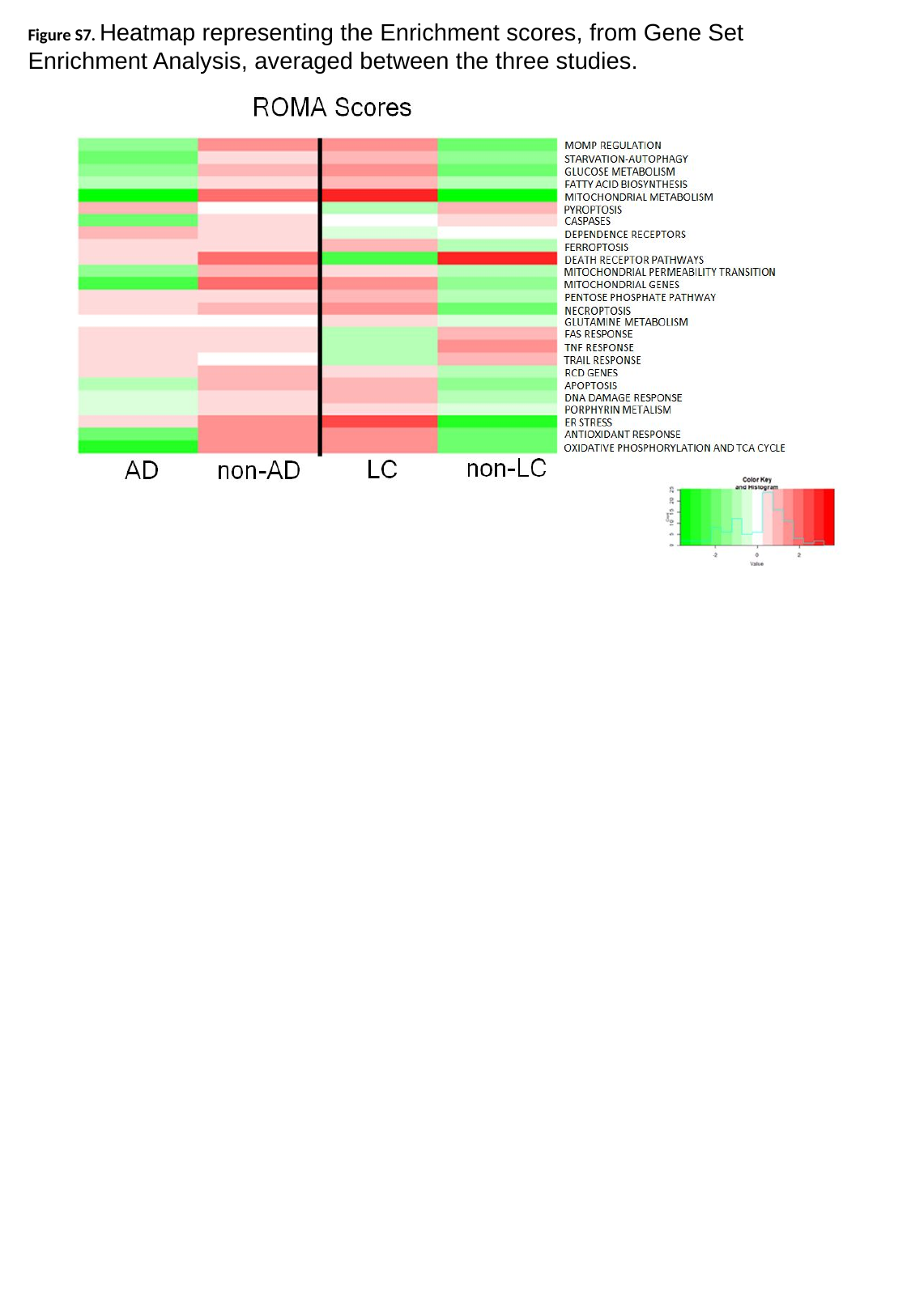

Figure S7. Heatmap representing the Enrichment scores, from Gene Set Enrichment Analysis, averaged between the three studies.

## Slide 12
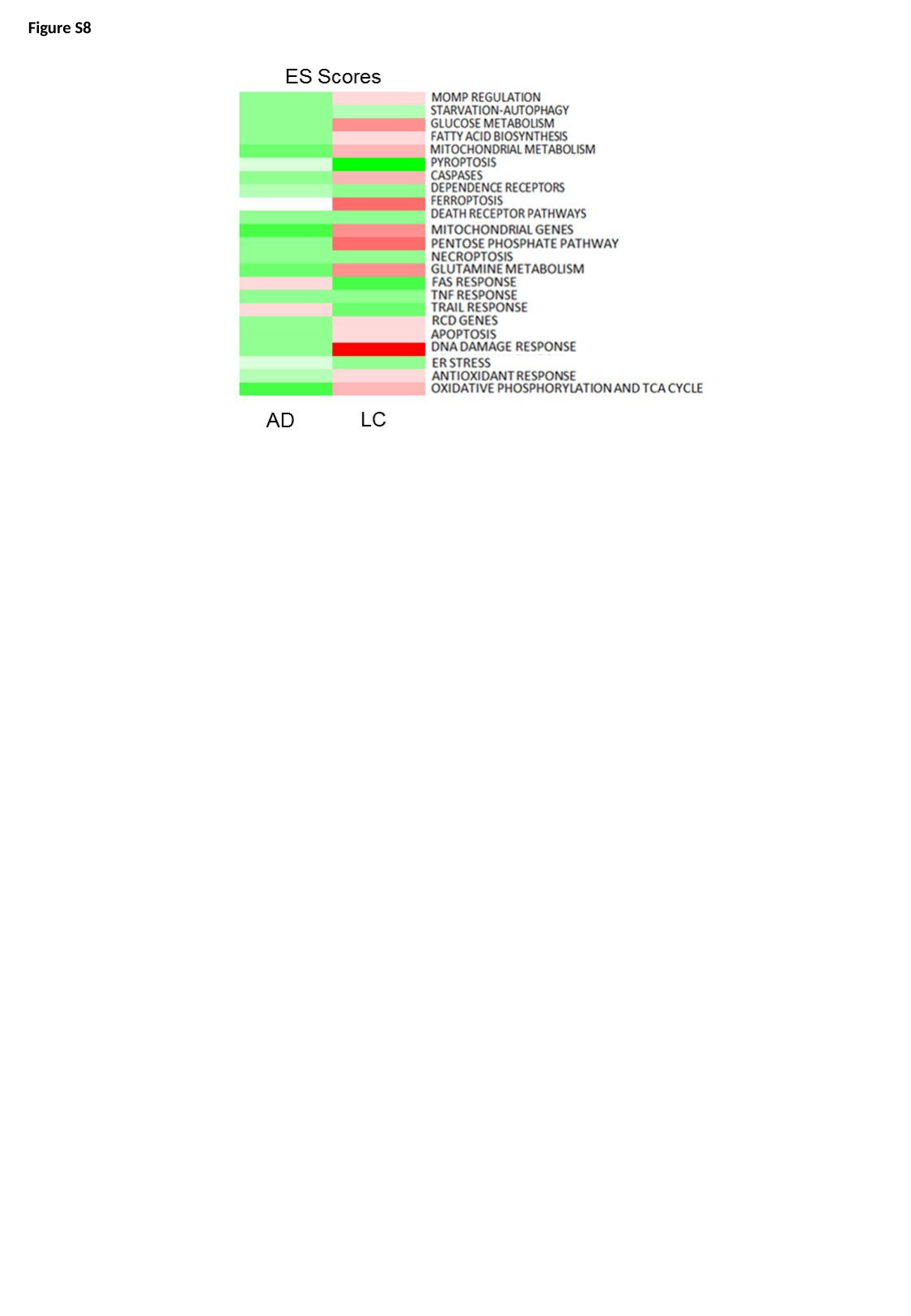

Figure S8
