# Supplementary material for: Comprehensive Map of the Regulated Cell Death Signaling Network: A Powerful Analytical Tool for Studying Diseases
Source: Cancers (Basel). 2020 Apr 17;12(4):990. doi: 10.3390/cancers12040990 (PMC7226067; doi:10.3390/cancers12040990)
Supplement: Supplementary file 1 [file cancers-12-00990-s001.zip › cancers-744176-supplementary-final/Table S2.docx]

**Table S2.** Comparison of gene content between pathways from KEGG and REACTOME databases to the Regulated Cell Death map. HUGO names were used as common IDs.

| **HUGO Names—Based Count: KEGG** | | | | | | |
| --- | --- | --- | --- | --- | --- | --- |
| **KEGG—pathway** | **RCD corresponding module** | **KEGG** | **ACSN** | **Common with ACSN** | **Not present in ACSN** | **Not present in KEGG** |
| Master map | _Master map | 956 | 891 | 423 | 533 | 468 |
| Glutathione metabolism | ANTIOXIDANT RESPONSE | 54 | 161 | 6 | 48 | 155 |
| Apoptosis | APOPTOSIS | 137 | 221 | 50 | 87 | 171 |
| Fatty acid biosynthesis | FATTY ACID BIOSYNTHESIS | 13 | 42 | 3 | 10 | 39 |
| Ferroptosis | FERROPTOSIS | 40 | 34 | 13 | 27 | 21 |
| Glycolysis Gluconeogenesis | GLUCOSE METABOLISM | 68 | 106 | 30 | 38 | 76 |
| Glutamate metabolism | GLUTAMINE METABOLISM | 4 | 20 | 4 | 0 | 16 |
| Citrate cycle (TCA cycle) | OXIDATIVE PHOSPHORYLATION AND TCA CYCLE | 30 | 185 | 22 | 8 | 163 |
| Oxidative phosphorylation | OXIDATIVE PHOSPHORYLATION AND TCA CYCLE | 133 | 185 | 69 | 64 | 116 |
| Pentose phosphate pathway | PENTOSE PHOSPHATE PATHWAY | 30 | 18 | 0 | 30 | 18 |
| Porphyrin and chlorophyll metabolism | PORPHYRIN METABOLISM | 42 | 18 | 11 | 31 | 7 |
| HIF-1 signalling pathway | RCD GENES | 100 | 137 | 17 | 83 | 120 |
| p53 signalling pathway | RCD GENES | 68 | 137 | 17 | 51 | 120 |
| mTOR signalling pathway | STARVATION-AUTOPHAGY | 151 | 167 | 34 | 117 | 133 |
| Autophagy - animal | STARVATION-AUTOPHAGY | 128 | 167 | 65 | 63 | 102 |
| Necroptosis | NECROPTOSIS + TNF RESPONSE + TRAIL RESPONSE + FAS RESPONSE | 165 | 154 | 52 | 113 | 102 |
| TNF signalling pathway | TNF RESPONSE + NECROPTOSIS + APOPTOSIS | 108 | 306 | 44 | 64 | 262 |

| **HUGO—Based Count: Reactome** | | | | | | |
| --- | --- | --- | --- | --- | --- | --- |
| **Reactome pathway** | **RCD corresponding module** | **Reactome** | **ACSN** | **Common with ACSN** | **Not present in ACSN** | **Not present in Reactome** |
| Master map | _Master map | 809 | 891 | 323 | 486 | 568 |
| Death Receptor Signalling | DEATH RECEPTOR PATHWAYS | 54 | 249 | 29 | 25 | 220 |
| Unfolded Protein Response | ER STRESS | 159 | 148 | 41 | 118 | 107 |
| Fatty acid metabolism | FATTY ACID BIOSYNTHESIS | 179 | 42 | 8 | 171 | 34 |
| Xylulose | GLUCOSE METABOLISM | 6 | 106 | 0 | 6 | 106 |
| Glucose metabolism | GLUCOSE METABOLISM | 96 | 106 | 33 | 63 | 73 |
| Glutamine metabolism | GLUTAMINE METABOLISM | 2 | 20 | 2 | 0 | 18 |
| TCA cycle and oxidative phosphorylation | OXIDATIVE PHOSPHORYLATION AND TCA CYCLE | 169 | 185 | 111 | 58 | 74 |
| Pentose phosphate pathway | PENTOSE PHOSPHATE PATHWAY | 15 | 18 | 0 | 15 | 18 |
| Metabolism of porphyrins | PORPHYRIN METABOLISM | 17 | 18 | 11 | 6 | 7 |
| mTOR signalling | STARVATION-AUTOPHAGY | 40 | 167 | 24 | 16 | 143 |
| Programmed Cell Death | APOPTOSIS + NECROPTOSIS | 178 | 284 | 49 | 129 | 235 |
