# Supplementary material for: Comprehensive Map of the Regulated Cell Death Signaling Network: A Powerful Analytical Tool for Studying Diseases
Source: Cancers (Basel). 2020 Apr 17;12(4):990. doi: 10.3390/cancers12040990 (PMC7226067; doi:10.3390/cancers12040990)
Supplement: Supplementary file 1 [file cancers-12-00990-s001.zip › cancers-744176-supplementary-final/Table S3.docx]

**Table 3.** Genes corresponding to copy number average equal or larger than 4 in the groups of ovarian cancer cases.

| **Differentiated** | | **Immunoreactive** | | **Mesenchymal** | | **Proliferative** | |
| --- | --- | --- | --- | --- | --- | --- | --- |
| ***Genes*** | ***Average*** | ***Genes*** | ***Average*** | *Genes* | *Average* | *Genes* | *Average* |
| CYC1 | 4.63 | ACER3 | 4.06 | BIRC7 | 4.09 | ACSS1 | 4.24 |
| DEPTOR | 4.68 | APH1A | 4.12 | COX6C | 4.05 | ACSS2 | 4.43 |
| DERL1 | 4.68 | ARNT | 4.12 | CYC1 | 4.67 | AIM2 | 4.14 |
| GPT | 4.63 | COX6C | 4.19 | DEPTOR | 4.52 | APH1A | 4.19 |
| GRINA | 4.63 | CREB3L4 | 4.06 | DERL1 | 4.67 | ARNT | 4.19 |
| MYC | 4.79 | CTSS | 4.12 | GPT | 4.67 | ATF6 | 4.19 |
| NDRG1 | 4.84 | CYC1 | 4.25 | GRINA | 4.52 | ATP5C1 | 4.05 |
| NDUFB9 | 4.68 | DEPTOR | 4.31 | MIR1.1 | 4.19 | ATP5E | 4.48 |
| SLC2A2 | 4.10 | DERL1 | 4.37 | MIR133A2 | 4.19 | BCL2L1 | 4.76 |
| TNFRSF11B | 4.53 | EIFB5 | 4.00 | MYC | 4.81 | BIRC7 | 4.48 |
| TNFSF10 | 4.05 | GPT | 4.25 | NDRG1 | 4.81 | C12orf5 | 4.38 |
| WISP1 | 4.84 | GRINA | 4.25 | NDUFB5 | 4.05 | CDKN1B | 4.24 |
|  | | MCL1 | 4.12 | NDUFB9 | 4.71 | COX4I2 | 4.76 |
|  |  | MYC | 4.56 | PIK3CA | 4.09 | CREB3L4 | 4.19 |
|  |  | NDRG1 | 4.50 | RRM2B | 4.05 | CSE1L | 4.29 |
|  |  | NDUFB5 | 4.19 | SLC2A2 | 4.00 | CSNK2A1 | 4.48 |
|  |  | NDUFB9 | 4.37 | TNFRSF11B | 4.57 | CTSS | 4.19 |
|  |  | NDUFC2 | 4.25 | TNFSF10 | 4.00 | DNAJB11 | 4.86 |
|  |  | NDUFS5 | 4.00 | WISP1 | 4.86 | DNM1L | 4.24 |
|  |  | NDUFS6 | 4.06 | YWHAZ | 4.05 | DUSP16 | 4.33 |
|  |  | PAK1 | 4.06 |  | | E2F1 | 4.48 |
|  |  | PIK3CA | 4.18 |  |  | E2F3 | 4.00 |
|  |  | RRM2B | 4.25 |  |  | EGLN1 | 4.00 |
|  |  | SDHA | 4.00 |  |  | EIF2AK2 | 4.00 |
|  |  | SLC2A2 | 4.50 |  |  | EIF2B4 | 4.00 |
|  |  | TFRC | 4.00 |  |  | EIF2B5 | 4.71 |
|  |  | TNFRSF11B | 4.31 |  |  | EIF2S2 | 4.48 |
|  |  | TNFSF10 | 4.44 |  |  | ENO2 | 4.48 |
|  |  | TRIT1 | 4.06 |  |  | FAIM | 4.05 |
|  |  | WISP1 | 4.50 |  |  | FASLG | 4.05 |
|  |  | YWHAZ | 4.12 |  |  | FBXO45 | 4.48 |
|  |  |  | |  |  | FH | 4.14 |
|  |  |  |  |  |  | FLAD1 | 4.19 |
|  |  |  |  |  |  | GABARAPL1 | 4.33 |
|  |  |  |  |  |  | GAPDH | 4.43 |
|  |  |  |  |  |  | GLUL | 4.09 |
|  |  |  |  |  |  | GSS | 4.43 |
|  |  |  |  |  |  | H2AFJ | 4.29 |
|  |  |  |  |  |  | HAX1 | 4.19 |
|  |  |  |  |  |  | HIST2H2AC | 4.19 |
|  |  |  |  |  |  | IDH3B | 4.38 |
|  |  |  |  |  |  | ING4 | 4.43 |
|  |  |  |  |  |  | ITCH | 4.48 |
|  |  |  |  |  |  | ITPR2 | 4.48 |
|  |  |  |  |  |  | KIAA0226 | 4.478 |
|  |  |  |  |  |  | LDHB | 4.19 |
|  |  |  |  |  |  | LMNA | 4.24 |
|  |  |  |  |  |  | MAP1LC3A | 4.48 |
|  |  |  |  |  |  | MCL1 | 4.19 |
|  |  |  |  |  |  | MIR1.1 | 4.48 |
|  |  |  |  |  |  | MIR133A2 | 4.48 |
|  |  |  |  |  |  | MIR16.2 | 4.43 |
|  |  |  |  |  |  | MIR199A2 | 4.09 |
|  |  |  |  |  |  | MIR214 | 4.09 |
|  |  |  |  |  |  | MIR23A | 4.09 |
|  |  |  |  |  |  | MYC | 4.14 |
|  |  |  |  |  |  | NCF2 | 4.09 |
|  |  |  |  |  |  | NDUFA9 | 4.38 |
|  |  |  |  |  |  | NDUFB5 | 4.81 |
|  |  |  |  |  |  | NDUFB7 | 4.33 |
|  |  |  |  |  |  | NDUFB9 | 4.09 |
|  |  |  |  |  |  | NDUFS2 | 4.19 |
|  |  |  |  |  |  | NFE2L2 | 4.00 |
|  |  |  |  |  |  | NLRP3 | 4.33 |
|  |  |  |  |  |  | NME7 | 4.09 |
|  |  |  |  |  |  | PFKFB3 | 4.09 |
|  |  |  |  |  |  | PIK3CA | 4.86 |
|  |  |  |  |  |  | PKLR | 4.24 |
|  |  |  |  |  |  | PLA2G4A | 4.00 |
|  |  |  |  |  |  | PPOX | 4.19 |
|  |  |  |  |  |  | PRKAB2 | 4.29 |
|  |  |  |  |  |  | PRKACA | 4.14 |
|  |  |  |  |  |  | RNF168 | 4.48 |
|  |  |  |  |  |  | RYR2 | 4.24 |
|  |  |  |  |  |  | SDHC | 4.19 |
|  |  |  |  |  |  | SHC1 | 4.19 |
|  |  |  |  |  |  | SLC2A2 | 4.86 |
|  |  |  |  |  |  | SLC2A3 | 4.38 |
|  |  |  |  |  |  | SRXN1 | 4.43 |
|  |  |  |  |  |  | TFB2M | 4.29 |
|  |  |  |  |  |  | TFRC | 4.48 |
|  |  |  |  |  |  | TNFRSF1A | 4.29 |
|  |  |  |  |  |  | TNFSF10 | 4.86 |
|  |  |  |  |  |  | TPI1 | 4.43 |
|  |  |  |  |  |  | TRIB3 | 4.48 |
|  |  |  |  |  |  | TRPC1 | 4.14 |
|  |  |  |  |  |  | TXNIP | 4.14 |
|  |  |  |  |  |  | UQCRFS1 | 4.00 |
|  |  |  |  |  |  | YWHAB | 4.24 |
