# Supplementary material for: Comprehensive Map of the Regulated Cell Death Signaling Network: A Powerful Analytical Tool for Studying Diseases
Source: Cancers (Basel). 2020 Apr 17;12(4):990. doi: 10.3390/cancers12040990 (PMC7226067; doi:10.3390/cancers12040990)
Supplement: Supplementary file 1 [file cancers-12-00990-s001.zip › cancers-744176-supplementary-final/Table S4.docx]

**Table S4. Gene set enrichment analysis scores.**

| **MODULE** | **MEAN ES SCORE AD** | **MEAN ES SCORE LC** |
| --- | --- | --- |
| MOMP_REGULATION | −0.36477934 | 0.07643606 |
| STARVATION_AUTOPHAGY | −0.333600753 | −0.254112823 |
| GLUCOSE_METABOLISM | −0.362907783 | 0.31258822 |
| FATTY_ACID_BIOSYNTHESIS | −0.38478577 | 0.0906174 |
| MITOCHONDRIAL_METABOLISM | −0.461833333 | 0.235647637 |
| PYROPTOSIS | −0.076380965 | −0.757206847 |
| CASPASES | −0.307371653 | 0.21491768 |
| DEPENDENCE_RECEPTORS | −0.27703751 | −0.318890747 |
| FERROPTOSIS | 0.03332813 | 0.45254224 |
| DEATH_RECEPTOR_PATHWAYS | −0.319047377 | −0.309892487 |
| MITOCHONDRIAL_GENES | −0.539613867 | 0.280868377 |
| PENTOSE_PHOSPHATE_PATHWAY | −0.349825643 | 0.429752847 |
| NECROPTOSIS | −0.369421087 | −0.306778717 |
| GLUTAMINE_METABOLISM | −0.4689607 | 0.296370293 |
| FAS_RESPONSE | 0.11738296 | −0.574906833 |
| TNF_RESPONSE | −0.32814571 | −0.359021753 |
| TRAIL_RESPONSE | 0.108715667 | −0.421699127 |
| RCD_GENES | −0.284315643 | 0.077840043 |
| APOPTOSIS | −0.320697753 | 0.067257243 |
| DNA_DAMAGE_RESPONSE | −0.34301881 | 0.83173605 |
| ER_STRESS | −0.145141807 | −0.384472043 |
| ANTIOXIDANT_RESPONSE | −0.18259398 | 0.114617577 |
| OXIDATIVE_PHOSPHOYLATION_AND_TCA_CYCLE | −0.55735252 | 0.257722337 |

Mean ES Scores of the six datasets used for ROMA analysis. ES: Enrichment Score, AD: Alzheimer’s disease, LC: Lung cancer.
